# Comparison of Different Types of Complications in the Phacoemulsification Surgery Learning Curve According to Number of Operations Performed

**DOI:** 10.4274/tjo.83788

**Published:** 2016-01-05

**Authors:** Mehmet Serhat Mangan, Eray Atalay, Ceyhun Arıcı, İbrahim Tuncer, Mustafa Değer Bilgeç

**Affiliations:** 1 Okmeydanı Education and Research Hospital, Ophthalmology Clinic, İstanbul, Turkey; 2 Kars State Hospital, Ophthalmology Clinic, Kars, Turkey; 3 İstanbul University Cerrahpaşa Faculty of Medicine, Department of Ophthalmology, İstanbul, Turkey; 4 Alfagöz Medical Center, İzmir, Turkey; 5 Osmangazi University Faculty of Medicine, Department of Ophthalmology, Eskişehir, Turkey

**Keywords:** Cataract surgery, learning curve, phacoemulsification

## Abstract

**Objectives:**

To compare the differences in intraoperative complications rates by the number of resident-performed sequential phacoemulsification surgeries.

**Materials and Methods:**

Preoperative and postoperative ophthalmological examination records and intraoperative data of 180 eyes of 140 patients who underwent cataract surgery by two residents between November 2009 and February 2012 were analyzed retrospectively. The data of 180 eyes were separated into 3 groups based on the number of operations performed: Group A (first 1-60 eyes), group B (61-120 eyes) and group C (last 121-180 eyes). The number of direct supervisor interventions and the rates of different types of complications were compared between the three groups.

**Results:**

The number of direct supervisor interventions was 45, 35 and 19 in group A, B and C, respectively. The number of complications anterior to the iris plane was 3, 4 and 12 in group A, B and C, respectively. The difference in the rate of complications between group B and C was statistically significant (p=0.029). The number of complications posterior to the iris plane was 6, 14 and 3 in group A, B and C, respectively. The difference in the rate of complications between the groups was statistically significant (p=0.042, p=0.004).

**Conclusion:**

This study provides insight into which types of complications might arise during the phacoemulsification training period. The trends in the rates of different complication types in clinics may be analyzed, and this analysis may be used to improve and modify phacoemulsification training programmes according to the needs of residents.

## INTRODUCTION

Phacoemulsification (phaco) is the most commonly performed surgery in ophthalmology and is a must for ophthalmology residents to learn during their training. Declines in intraoperative complications, number of direct supervisor interventions required and operation time are measures of the learning curve of resident phaco surgery.^1,2,3,4,5^ According to the many studies performed in this field, operation times and complication rates are reported to change as the resident gains experience in cataract surgery.^1,2,3,4,5,6,7^ In several studies, an inverse relationship was observed between the complication rate and residency year.^1,2,6^

One of these studies examined complication rates of residents according to their residency year and although it reports no significant association with overall intraoperative complications, vitreous loss did occur more frequently in surgeries performed by second-year residents.^1^ Another study also found no significant difference between second- and third-year resident phaco surgeries.^7^ From the residents’ point of view, capsulorhexis was the most difficult step for second-year residents and the nuclear emulsification procedure was the most difficult step for third-year residents according to one study.^8^

Recent studies have compared complication rates of residents by their year of residency, but it is well accepted that the phaco learning curve is directly correlated with the number of operations performed and not the residency year. The trend in the rate of different complications in resident-performed phaco surgery might reveal clues about which stages of phaco surgery are most challenging for residents at various surgical skill levels. Analysis of this trend may yield a new perspective in resident phaco training. The purpose of this study was to compare the differences in intraoperative complication rates by the number of surgeries residents had performed. 

## MATERIALS AND METHODS

The setting of this retrospective study was a tertiary care university hospital. Preoperative and postoperative ophthalmological examination records and intraoperative data of 180 eyes of 140 patients (67 female, 73 male) who underwent cataract surgery at İstanbul University, Cerrahpaşa Faculty of Medicine were analyzed. The study followed the tenets of the Declaration of Helsinki. All patients signed a consent form after receiving an explanation of the nature and possible consequences of the procedure.

Surgeries were performed between November 2009 and February 2012 by two residents. Before their direct attendance to phaco surgeries, these residents had attended several lectures and observed nearly 30 phaco surgeries. The residents had not performed any extracapsular cataract extraction surgery prior to phaco surgery training. Patients were preoperatively informed about the resident contribution to his/her surgery and informed consent was signed by each patient. Cataract surgery was performed by residents under the supervision of a qualified cataract surgeon. Patients were selected randomly from the outpatient clinic with no specific criteria in terms of case difficulty excluding traumatic cases, patients with zonular dehiscence and cases necessitating combined procedures.

The phacoemulsification procedure was performed under topical or retrobulbar anesthesia according to the patient’s status and the surgeon’s decision. A 3.2 mm clear corneal incision was used and two side ports were created for bimanual phacoemulsification. A continuous curvilinear capsulorhexis was performed with the help of trypan blue when needed. The lens was emulsified using the “divide and conquer” technique in all cases. The surgery was concluded with the insertion of an injectable hydrophobic acrylic intraocular lens (IOL) in the bag or within the ciliary sulcus when possible, followed by the administration of intracameral antibiotics. Direct supervisor intervention was required in surgeries in which complications could not be managed by the resident surgeon.

Analysed data included age, sex, ocular comorbidities, preoperative and postoperative best corrected visual acuity (BCVA), date of operation, operative complications, attending cataract and resident surgeon, number of cases performed by the resident, and number of operations which necessitated direct supervisor intervention. Operative complications included anterior capsule tears, posterior capsule rupture, wound burns, dropped nucleus or nuclear fragments, vitreous loss, Descemet’s membrane detachments and corneal edema lasting for more than 10 days postoperatively. Operative complications were divided into two categories with respect to their anatomical position: complications anterior to the iris plane included wound burns, Descemet’s membrane detachments, and corneal edema lasting for more than 10 days; complications posterior to the iris plane included anterior capsule tears, posterior capsule rupture (with/without vitreous loss), zonular dialysis and dehiscence, and dropped nucleus or nuclear fragments. Each complication for each surgery was separately recorded and grouped according to its anatomic position. All data of the 180 eyes were grouped into three categories according to the number of operations previously performed by the resident. Chi-square test for independence was used to compare the rates of different types of complications observed in the groups. Statistical Package for the Social Sciences 16.0 for Windows was used for statistical calculations.

## RESULTS

A total of 180 eyes of 140 patients (73 male, 67 female) underwent phacoemulsification surgery by 2 residents under the supervision of a qualified cataract surgeon. The data of the 180 eyes were grouped into 3 categories with 60 operated cases in each group. Groups A, B and C consisted of the data from surgeries 1-60, 61-120, and 121-180, respectively, performed by the residents.

Mean (standard deviation) patient age at the time of surgery was 67.3 (6.9) years (range, 52-81 years). Direct supervisor intervention was recorded in 45 (75%) surgeries in group A, 35 (58.3%) surgeries in group B and 19 (16.6%) surgeries in group C.

Of the 180 operations performed, complications were observed in 42 (23.3%) (Table 1). The total numbers of complications in groups A, B and C were 9, 18, and 15, respectively. There was no statistically significant difference in total complication rates among the groups (p=0.141).

Posterior capsule rupture with or without vitreous loss was observed in 15 (8.8%) eyes, 9 of which were in group B. The IOL could be implanted into the sulcus in 14 eyes where there was sufficient capsular support, and 1 eye underwent scleral-fixated IOL implantation surgery.

Dropped nucleus or nuclear fragments occurred in 2 cases, both in group B. Synchronous pars plana vitrectomy was performed for these cases; the IOL could be implanted into the sulcus in 1 patient, while the other patient required scleral-fixated IOL implantation surgery.

The number of complications anterior to the iris plane was 3, 4 and 12 in groups A, B and C respectively. The difference in the rate of complications between group B and C was statistically significant (p=0.029). The number of complications posterior to the iris plane was 6, 14 and 3 in groups A, B and C respectively. The difference in the rate of complications between the groups was statistically significant (p=0.042, p=0.004).

## DISCUSSION

The incidence of complications in resident phacoemulsification surgeries ranges from 1.8% to 27.4% in different studies.^9^ The overall rate of complications in this study was 23.3% and is consistent with other studies. There were no cases of endophthalmitis in our study. The rate of major complications such as posterior capsule rupture and vitreous loss may affect surgical and visual outcomes. Posterior capsule rupture with or without vitreous loss occured in 15 cases (8.8%). This rate is comparable to rates reported in the literature (2.5-14.7%).^3,9,10,11,12,13,14,15^

Sixty percent of the posterior capsule ruptures occured in group B. The reason for the low complication rates in group A could be the high intervention rates (75% of surgeries) by the supervising cataract surgeon. The complication rates increase as the intervention rates of the supervising cataract surgeon decrease. Although intervention rates decreased in group C, vitreous loss occurred in only 3 cases. This finding may suggest that the resident phacoemulsification learning curve was assessed with regard to total number of cases, with a break point of 60 cases identified in our study. This finding is similar to a previous study by Randleman et al.,^6^ who reported a break point of 80 cases. The Residency Review Committee of the Accreditation Council for Graduate Medical Education16 has recently increased the minimum number of cataract procedures performed by the resident as primary surgeon from 45 to 86. Thus, individual variations are common not only in surgical skill but also in surgical volume among residents of the same level.

Several previous studies reported that residency year was not a risk factor for intraoperative complications.^1,7^ Therefore, in our study all data of the 180 eyes were grouped into 3 categories according to the number of operations performed by the resident rather than residency year.

Complications during phaco surgery primarily occur due to mechanical trauma by the phaco probe or the application of excessive ultrasound energy to the ocular tissues. The rise of complications posterior to the iris plane in group B could be explained by the learning curve for nuclear emulsification. The occurrence of dropped nucleus or nuclear fragments in group B can also be interpreted as a consequence of this learning curve. Woodfield et al.^7^ supports this idea as posterior capsule ruptures were more frequently observed in second-year residents in their study.

The current study also found a low rate of dislocated lens fragments in the vitreous: 2 eyes (1.11%). The reported risk of requiring pars plana vitrectomy to remove lens fragments dislocated during cataract surgery ranges from 0.2% to 1.68% in the literature.^12,17,18^

While the rates of complications posterior to the iris dropped significantly between group B and C, complications anterior to the iris plane peaked in group C. This could be explained by the rise in posterior capsule ruptures in group B, which might have influenced the resident to utilize phacoemulsification more anteriorly in order to avoid posterior capsule related complications. Consequently, wound burn, Descemet’s membrane detachment and corneal edema lasting longer than 10 days were observed more frequently in group C. This data is also consistent with previous studies. Woodfield et al.^7^ found capsule tears more frequently in second-year resident surgeries and wound burns more frequently in third-year resident surgeries.

The rate of supervisor intervention is also a measure in the learning curve of resident phaco surgery.^1,3^ Direct supervisor intervention was required in 90 of the cases, and the rest were completed exclusively by the residents in this study. The rate of direct supersivor requirement (50%) is similar to previous studies. Lee et al.^1^ reported a completion rate of 47% and Dooley and O’Brien3 reported a completion rate of 42% in their studies.

In an article published in Turkey by Erdoğan et al.^19^ related to the phacoemulsification learning period, the rates of posterior capsule rupture and dropped nucleus were 10.6% and 2.7%, respectively. These rates are similar to those in our study.

Limitations of this study include the retrospective nature of the study and the relatively small numbers of surgeons and surgical cases analyzed. Prospective validation of our findings will be necessary.

## CONCLUSION

Phacoemulsification is a challenging technique to learn and to teach due to its small error margins. Residents face nearly all complications of phaco surgery in the learning curve period. These complications show a rise when the resident starts to take more action during the surgery. This is one of the few studies which report the trend in the rates of different complications of resident phaco surgery according to number of operations performed, and it provides some insight into which types of complications might arise in the phaco training period. Trends in the rates of different complication types in clinics may be analyzed, and this analysis may be used to improve and modify phaco training programmes according to the needs of residents.

## Ethics

Ethics Committee Approval: It was taken, Informed Consent: It was taken.

Peer-review: External and Internal peer-reviewed.

## Figures and Tables

**Table 1:**
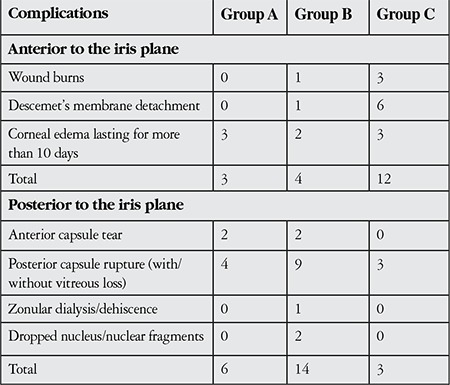
Distribution of complications among groups

## References

[1] Lee JS, Hou CH, Yang ML, Kuo JZ, Lin KK (2009). A different approach to assess resident phacoemulsification learning curve: analysis of both completion and complication rates. Eye (Lond).

[2] Tayanithi P, Pungpapong K, Siramput P (2005). Vitreous loss during phacoemulsification learning curve performed by third-year residents. J Med Assoc Thai.

[3] Dooley IJ, O’Brien PD (2006). Subjective difficulty of each stage of phacoemulsification cataract surgery performed by basic surgical trainees. J Cataract Refract Surg.

[4] Hosler MR, Scott IU, Kunselman AR, Wolford KR, Oltra EZ, Murray WB (2012). Impact of resident participation in cataract surgery on operative time and cost. Ophthalmology.

[5] Wiggins MN, Warner DB (2010). Resident physician operative times during cataract surgery. Ophthalmic Surg Lasers Imaging.

[6] Randleman JB, Wolfe JD, Woodward M, Lynn MJ, Cherwek DH, Srivastava SK (2007). The resident surgeon phacoemulsification learning curve. Arch Ophthalmol.

[7] Woodfield AS, Gower EW, Cassard SD, Ramanthan S (2011). Intraoperative phacoemulsification complication rates of second- and third-year ophthalmology residents a 5-year comparison. Ophthalmology.

[8] Prakash G, Jhanji V, Sharma N, Gupta K, Titiyal JS, Vajpayee RB (2009). Assessment of perceived difficulties by residents in performing routine steps in phacoemulsification surgery and in managing complications. Can J Ophthalmol.

[9] Carricondo PC, Fortes ACFM, Mourao PC, Hajnal M, Jose NK (2010). Senior resident phacoemulsification learning curve. Arq Bras Oftalmol.

[10] Johansson B, Lundström M, Montan P, Stenevi U, Behndig A (2009). Capsule complication during cataract surgery: Long-term outcomes: Swedish Capsule Rupture Study Group report 3. J Cataract Refract Surg.

[11] Blomquist PH, Rugwani RM (2002). Visual outcomes after vitreous loss during cataract surgery performed by residents. J Cataract Refract Surg.

[12] Bhagat N, Nissirious N, Potdevin L, Chung J, Lama P, Zarbin MA, Fechtner R, Guo S, Chu D, Langer P (2007). Complications in resident-performed phacoemulsification cataract surgery at New Jersey Medical School. Br J Ophthalmol.

[13] Rutar T, Porco TC, Naseri A (2009). Risk factors for intraoperative complications in resident- performed phacoemulsification surgery. Ophthalmology.

[14] Allinson RW, Metrikin DC, Fante RG (1992). Incidence of vitreous loss among third year residents performing phacoemulsification. Ophthalmology.

[15] Yulan W, Yaohua S, Jinhua T, Min W (2013). Step-by-step phacoemulsification training program for ophthalmology residents. Indian J Ophthalmol.

[16] (Accessed February 2007). Accreditation Council for Graduate Medical Education. Ophthalmology resident operative minimum requirements.

[17] Gimbel HV, Sun R, Ferensowicz M, Anderson Penno E, Kamal A (2001). Intraoperative management of posterior capsule tears in phacoemulsification and intraocular lens implantation. Ophthalmology.

[18] Schwartz SG, Holz ER, Mieler WF, Kuhl DP (2002). Retained lens fragments in resident performed cataract extractions. CLAO J.

[19] Erdoğan H, Toker Mİ, Arıcı MK, Özdemir Z, Topalkara A (2002). Evaluation of phacoemulsification results in learning period. Turk J Ophtalmol.

